# Application of Narrow-Bore HPLC Columns in Rapid Determination of Sildenafil Citrate in Its Pharmaceutical Dosage Forms

**Published:** 2012

**Authors:** Razieh Ghodsi, Farzad Kobarfard, Sayyed Abbas Tabatabai

**Affiliations:** a *DepartmentofPharmaceuticalChemistry, SchoolofPharmacy, ShahidBeheshtiUniversityofMedicalSciences, Tehran, Iran*; b *PhytochemistryResearchCenter, ShahidBeheshtiUniversityofMedicalSciences, Tehran, Iran.*

**Keywords:** Sildenafil citrate, Determination, HPLC, Narrow-bore C18 column, Pharmaceutical dosage forms

## Abstract

A special type of silica-based columns has been recently introduced into the market which is called narrow-bore columns. They have lower internal volume than the standard high-performance liquid chromatography (HPLC) columns and thus reduce the solvent consumption by almost 80%.

A simple, accurate and environmentally friendly reversed phase- HPLC (RP-HPLC method) which could be used in fast and high throughput analyses has been developed for the purpose of determining the sildenafil in bulk and pharmaceutical dosage forms, using narrow-bore C_18_ column (50 × 3.2 mm, 5 µm particle size) in isocratic mode, with mobile phase comprising of buffer (pH = 3) and acetonitrile in the ratio of 75:25 v/v.

The flow rate was 0.7 mL/min and the detection was monitored through Ultraviolet detector (UV detector) at 292 nm. Clonazepam was used as the internal standard and the run time was 4 min.

The proposed method has permitted the quantification of sildenafil over the linearity in the range of 30-4000 ng/mL and its percentage recovery was found to be 99-105%. Limit of quantitation (LOQ) is determined as 30 ng/mL. The intra-day and inter-day precisions were found 1.2-2.2% and 1.56-3.4% respectively. The solvent consumption was 2.8 mL per sample of which ca 0.7 mL was acetonitrile.

This study shows that the application of narrow-bore column instead of the conventional reversed phase column in HPLC analyses has the advantages of shorter run time and less organic solvent consumption. This method is highly sensitive with excellent recoveries and precision and there is no need for special column and pre-column or post-column treatment of the sample. Moreover, the method is free from interference by common additives and excipients, suggesting applications in routine quality control analyses.

## Introduction

**Figure 1:**
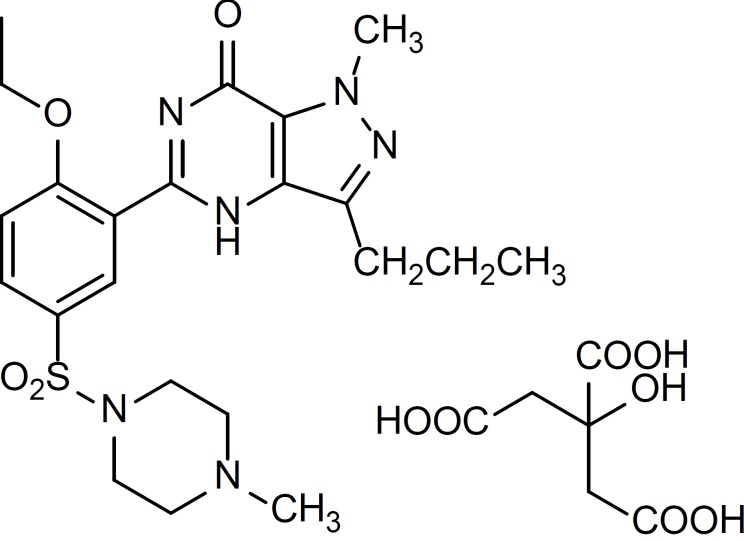
Structure of Sildenafil Citrate (SC).

Sildenafil (Figure 1) is a potent and selective inhibitor of cyclic Guanosine Monophosphate (cGMP) (type V) specific phosphodiesterase which is capable of enhancing the relaxation of penile corpus cavernosum and therefore has the potential to improve penile erectile function (1). No official method is currently available for the determination of sildenafil in pharmaceutical preparations and only few methods have been reported in the literatures (2-7).

Chromatographic separation is one of the essential and powerful components of the most quantitative analyses and high-performance liquid chromatography (HPLC) is currently the most versatile tool which satisfies the needs for an optimum separation. However, the high cost of HPLC grade solvents and the lengthy process of separation in some cases have always been one of the main concerns of analytical chemists. On the other hand, the recent growing concerns about the environmental problems caused through the utilization of large amounts of organic solvents in different fields of science and industries including analytical chemistry calls for faster, simpler and less solvent-demanding methods.

A special type of silica-based columns has been recently introduced into the market which is called narrow-bore columns. They have lower internal volume than the standard HPLC columns and thus reduce the solvent consumption by almost 80%.

Narrow-bore columns operate at lower flow rates (0.1-0.5 mL/min) with much reduced peak volumes, which results in reduced solvent consumption and increased sensitivity (8-10).

In the present research, narrow-bore column has been used to develop an environmentally friendly method for the analysis of sildenafil which could be used in fast and high throughput analysis of sildenafil in its pharmaceutical preparations and this method is more sensitive and rapid than the methods that have been reported in the literatures (2-6).

## Experimental

All solvents were of analytical grade and purchased from Merck (Darmstadt, Germany). Water was prepared by a Millipore Milli-Q Plus Water Purification System.

Sildenafil citrate and clonazepam were donated by Marham Daru pharmaceutical company (Tehran, Iran). Sildenafil tablets were a product of Marham Daru pharmaceutical Co.; each tablet was labeled to contain 100 mg sildenafil citrate.

*Chromatographic system*

Chromatographic analysis was performed on a Merck-Hitachi HPLC system consisted of a binary pump (L-7100), autosampler (L-7250) and Ultraviolet detector (UV detector) (L-7420) operated at 292 nm. Separation was carried out on a reversed-phase narrow-bore C18 column (50 × 3.2 mm, 5 µm particle size, Capital HPLC).

The mobile phase was a mixture of phosphate buffer (20 mM, pH = 3.0): acetonitrile (75:25 v/v) and it was filtered before being used through a 0.45 µm nylon-membrane filter (Waters, USA) and degassed under vacuum.

The flow rate was 0.7 mL/min and Clonazepam was used as internal standard and run the time was 4 min. Chromatography was conducted at ambient temperature and analyte to internal standard peak area ratios was used for quantification of sildenafil.

*Standard solutions*

The stock standard solution of sildenafil citrate (SC) at the concentration of 10 µg/mL was prepared by dissolving the required amount of SC in mobile phase and stored at 4^°^C. Working standard solutions of SC (30-4000 ng/mL) and clonazepam as the internal standard (500 ng/mL) were prepared by diluting the standard stock solution with the appropriate amounts of mobile phase.

To construct the calibration curve, five replicates (100 µL) of each peak area ratios of SC to Clonazepam were plotted against SC concentrations in standard solutions.

Quality control (QC) samples were prepared at three concentrations (50, 200, 2000 ng/mL). Separate stock standard solutions of SC were used for the preparation of calibration standard solutions and QC samples.

The calibration standards and QC samples were prepared freshly every day and found to be stable during the time required for the analysis.

*Assay procedure for dosage forms*

Twenty tablets were used to calculate the average weight of a tablet and then finely powdered. An accurately weighed amount of this powder, equivalent to 100 mg of sildenafil citrate, was transferred into a 200 mL volumetric flask and 150 mL of mobile phase was then added and the mixture was sonicated for 15 min and the volume was adjusted to 200 mL with the mobile phase. The sample solutions were then filtered and 1 mL of the filtrate was transferred to a 100 mL volumetric flask and brought to the volume with mobile phase.

**Figure 2:**
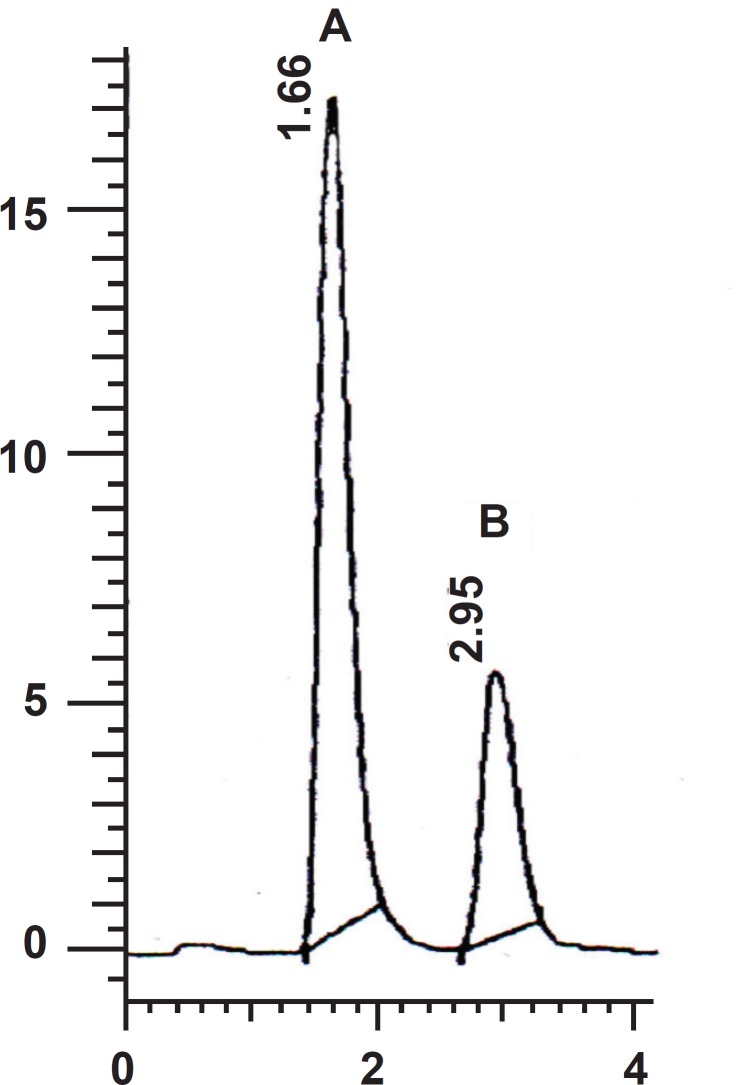
Representative chromatogram for (A) SC 2 µg/mL, (B) Clonazepam (500 ng/mL) as internal standard

In order to conduct the content uniformity of the tablets, each tablet was transferred into 200 mL separate volumetric flasks and then treated the same way described above.

One mL of the resulted solution and one mL of clonazepam solution (10 µg/mL) were transferred to a 10 mL volumetric flask and the volume was adjusted to 10 mL. Finally, 100 µL of this diluted sample was injected into the HPLC system.

## Results and Discussion

Chromatography was conducted on a narrow-bore column and the chromatographic conditions were optimized to obtain the proper retention times for sildenafil and clonazepam.

The effects of the percentage of organic solvent and the concentration of phosphate buffer in the mobile phase on the retention times of sildenafil and clonazepam were investigated. Acetonitrile was preferred over methanol as the organic phase since it resulted in a better peak shape and resolution.

The dependence of log k^′^ (capacity factor) values of analyte and internal standard on acetonitrile percentage was nearly linear.

Utilization of more than 50% acetonitrile caused the early elution of the analyte peak and acetonitrile concentrations lower than 20% caused seriously delayed elution and peak broadening. The effect of phosphate buffer concentration on the log k′-values of the compounds was evaluated over the concentration range of 5 to 50 mM and the concentration of 20 mM showed the best result.

The optimum wavelength for detection was 292 nm at which the best detector responses for both sildenafil and clonazepam were obtained. Figure 2 shows a representative chromatogram in this analysis.

Under the optimum chromatographic conditions, tailing and asymmetry factors were 1.12 and 1.14 for sildenafil and 1.09 and 1.12 for clonazepam respectively.

**Figure 3:**
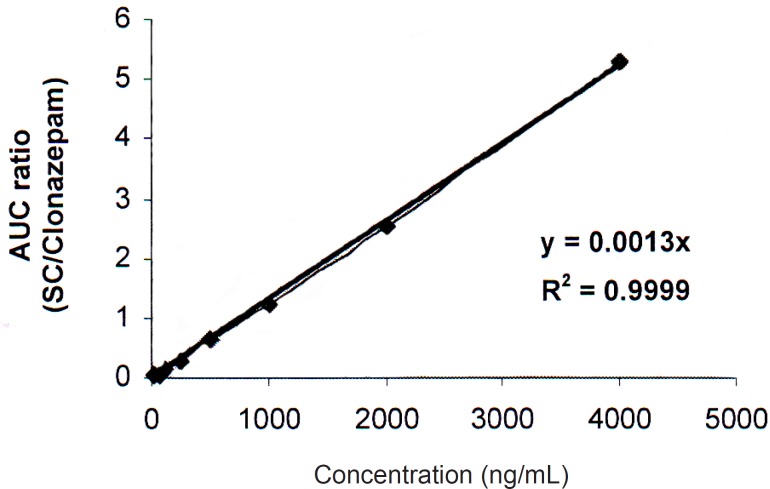
Calibration curve of SC

*Calibration curve, precision and accuracy*

For calibration, sildenafil citrate standard solutions were analyzed in triplicate in six analytical runes. Linear relationships were obtained between the analyte-to-internal standard peak-area ratios and the corresponding concentrations (correlation coefficient = 0.9999).

Correlation coefficients invariably exceeded 0.9995.

Calibration curve was linear over the concentration range of 30-4000 ng/mL (Figure 3). The limit of quantitation (LOQ) is determined as 30 ng/mL. One-way analysis of variance (ANOVA) was used to assess intra- and inter-day assay precision. Intra-day and Inter-day relative standard deviations (RSDs) were 0.36-1.62% and 1.53-1.73% respectively. The accuracy of the method recovery was calculated 99.43-101.60% (Tables 1 and 2).

*Application of the method to the analysis of commercial formulations*

The developed method was successfully utilized to determine the SC content of 12 different tablets and was proved to be suitable for the routine quality control analyses without interference from the excipients and additives such as starch, glucose, lactose and magnesium stearate.

The method was suitable for the content uniformity testing, in which many assay on individual tablets are required. Five different lots of commercially available tablets containing sildenafil citrate were analyzed using the developed method. Therefore, this method can be used for accurate and precise quantification of sildenafil citrate in pharmaceutical dosage forms. The aim of the present study was to evaluate the efficiency of narrow-bore C-18 column for the high-throughput analysis of sildenafil citrate in its pharmaceutical dosage forms.

The chromatographic parameters assessed in this study included capacity factor, resolution, peak width, selectivity and peak form.

The run time in our experiment was 4 min.

When the flow rate was 0.7 mL/min, the solvent consumption in our experiment was 2.8 mL per sample of which ca 0.7 mL was acetonitrile. This is much less than what would be needed on a 4.6 mm column.

This study shows that the application of narrow-bore column instead of the conventional reversed phase column in HPLC analyses has the advantages of shorter run time and less organic solvent consumption.

Chromatographic performance assessed in terms of selectivity, resolution, symmetry and tailing were easily optimized on this type of column.

We encourage the routine use of narrow-bore columns in high throughput analysis of pharmaceutical substances in their different dosage forms.

**Table 1:** Intra-day precision and accuracy of quality control samples for the determination of SC.

**Actual concentration (μg/mL)**	**Calculated concentration (μg/mL)**	**Accuracy %**	**Precision% (R.S.D)**
0.175	0.174	99.43	0.71
1.750	1.778	101.60	1.62
3.500	3.487	99.63	0.36

**Table 2:** Inter-day precision and accuracy of quality control samples for the determination of SC.

**Actual concentration (μg/mL)**	**Calculated concentration (μg/mL)**	**Accuracy %**	**Precision% (R.S.D)**
0.175	0.178	101.71	1.60
1.750	1.723	98.46	1.53
3.500	3.561	101.74	1.73

## Conclusions

The developed method for the determination of SC by narrow-bore C18 column with UV detection yielded excellent recoveries and precision. The method was shown to be simple, rapid and reproducible. The validity of the method is well-demonstrated by the analysis of SC tablets from different manufacturers. This method is highly sensitive and there is no need for special column and pre-column or post-column treatment of the sample. Moreover, the method is free from interference by common additives and excipients, suggesting the applications in the routine quality control analyses.
